# Adequate vitamin D levels in a Swedish population living above latitude 63°N: The 2009 Northern Sweden MONICA study

**DOI:** 10.3402/ijch.v74.27963

**Published:** 2015-05-13

**Authors:** Anna Ramnemark, Margareta Norberg, Ulrika Pettersson-Kymmer, Mats Eliasson

**Affiliations:** 1 Geriatric Medicine, Department of Community Medicine and Rehabilitation, Umeå University, Umeå, Sweden; 2 Epidemiology and global health, Department of Public Health and Clinical Medicine, Umeå University, Umeå, Sweden; 3 Clinical Pharmacology, Department of Pharmacology and Clinical Neuroscience, Umeå University, Umeå, Sweden; 4 Department of Public Health and Clinical Medicine, Sunderby Research Unit, Umeå University, Umeå, Sweden; 5 Arctic Research Centre (Arcum), Umeå University, Umeå, Sweden

**Keywords:** hydroxyvitamin D levels, vitamin D insufficiency/deficiency/status, population study, observational (cohort) design, age, gender

## Abstract

**Background:**

Even though vitamin D is mainly produced by exposure to sunlight, little is known regarding vitamin D levels in populations living in sub-Arctic areas with little or no daylight during winter.

**Objective:**

We describe distributions of vitamin D3 and the prevalence of adequate levels in a population living above 63°N.

**Design:**

We sampled 1,622 randomly selected subjects, aged 25–74 years, between January and May, 2009, as part of the Northern Sweden MONICA study (69.2% participation rate). By using HPLC, 25(OH) vitamin D3 was analysed. Levels used for definitions were deficient, D3<25 nmol/l (<10 ng/ml); insufficient, D3 25–49.9 nmol/l (10–20 ng/ml); and adequate, D3≥50 nmol/l (20 ng/ml).

**Results:**

Mean (median) level of vitamin D3 was 65.2 (63.6) nmol/l in men and 71.0 (67.7) nmol/l in women. Adequate levels were found in 79.2%, more often in women (82.7%) than in men (75.6%). Only 0.7% of the population were vitamin D3–deficient but 23.1% of men and 17.1% of women had insufficient levels. Levels of vitamin D3 increased with age and insufficient status was most common among those aged 25–34 years, 41.0% in men and 22.3% in women.

If subjects using vitamin D-supplementation are excluded, the population level of D3 is 1–2 nmol/l lower than in the general population across sex- and age groups. There were no differences between the northern or the southern parts, between urban or rural living or according to educational attainment. Those subjects born outside of Sweden or Finland had lower levels.

**Conclusion:**

The large majority living close to the Arctic Circle in Sweden have adequate D3 levels even during the second half of the dark winter. Subjects with D3 deficiency were uncommon but insufficient levels were often found among young men.

Vitamin D, primarily produced by skin exposure to sunlight, is required for optimal bone health. Deficiency contributes to osteoporosis, reduces muscular function and increases risks for falls and fractures. Beyond this, much attention has focused on vitamin D and its expected consequences for general health, survival and specific diseases, recently summed up in 2 systematic reviews (1,2). Although many diseases are more common and mortality higher (3) with low levels of vitamin D, evidence for causality is often lacking. The US Preventive Service Task Force in 2015 concluded that treatment with vitamin D may reduce mortality but only in institutionalized elderly, and risk for falls, but not fractures (2). A meta-analysis from 2014 found a small reduction of all-cause mortality after vitamin D supplementation among older adults (1).

Living in the northern part of the world, even above the Arctic Circle, provides little or no sun exposure during the winter months. For some diseases, a gradient has been identified between prevalence of vitamin D deficiency and latitude, but results are ambiguous (4). In Sweden, a marked increase in the incidence of hip fracture for every degree increase in latitude (5) underscores the possibility of an interaction between high latitude, low vitamin D and ill health, although the etiology of hip fractures include many other factors.

In previous Nordic studies of the domestic population, mean values are generally adequate, the seasonal variation is significant and the prevalence of vitamin D deficiency is low (6–15) However, in specific populations such as immigrants, both insufficiency and deficiency are significantly more common and might affect both health and quality of life (16,17). Direct comparisons of contemporary vitamin D levels in the population across Europe are scarce but a large multicentre study from 2013 reported higher vitamin D levels with higher latitude in Europe (18). A review on vitamin D in the Arctic population underscored the importance of traditional foods to avoid deficiency of vitamin D (19).

Many reports describe the situation in the 1990s (7,8,11–15,20) although few reports include men (8,12,13,20), and some are based on case-control studies and not cohort studies (6,21,22). Other shortcomings are small studies and limited age spans. Since sun exposure is proposed to be the major determinant for adequate levels, it would be important to study the northernmost parts of the Scandinavian countries and Russia during the winter months when the sun does not rise above the horizon (6,22). A recent report from Greenland describes increasing vitamin D deficiency with transition to modernized foods (23). Large cohort studies including both adult men and women could answer if modern day living with travelling for sun holidays, using solariums and taking vitamin D supplements would counterbalance the ever dark winter.

The aim of this study was to evaluate the levels of vitamin D in a representative adult population of northern Sweden (above latitude 63°N) in 2009.

## Material and methods

### Survey participants

**Fig. 1:**
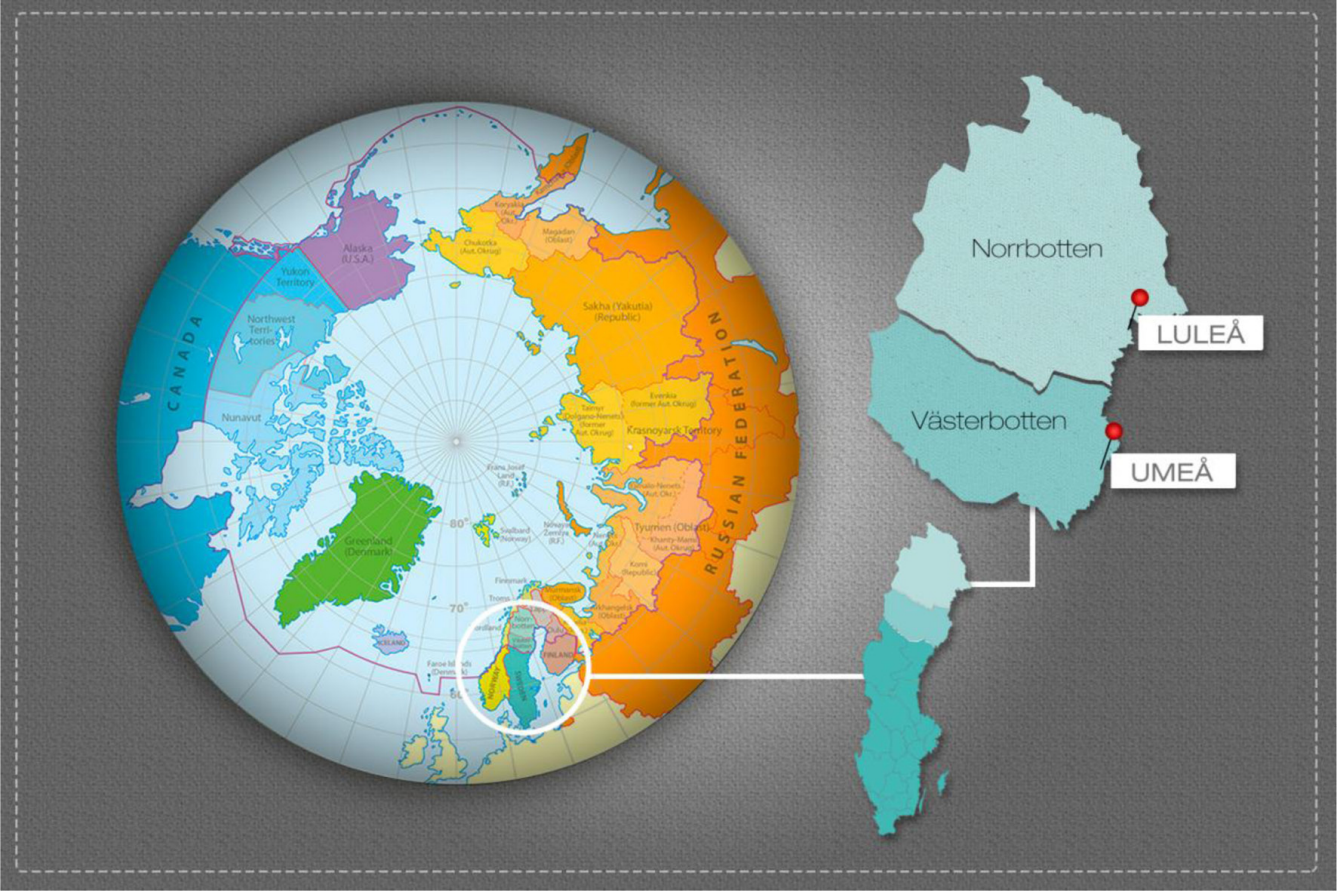
Norrbotten and Västerbotten counties and the 2 residential centres of Umeå and Luleå in northern Sweden.

We used data from the Northern Sweden MONICA study, a population-based survey conducted in the 2 northern counties of Sweden with a target population of 312,000 inhabitants aged 25–74 years. In these counties, the 2 major population centres are Umeå at 63.8° N and Luleå at 65.6° N (Fig. 1). In total, 2,500 subjects aged 25–74 years were randomly selected from population registers and stratified for age and gender. Details of sampling and selection have been presented and data on non-participants have been published (24,25). The examinations were accomplished in January to April 2009. In total 1,729 subjects participated (69.2%).

### Measurement and lab procedures

Details regarding examination and definition have previously been extensively described (25). Questionnaires covering educational achievement, country of birth, type of residential area (26) and current medication were administered.

### Blood samples and lab

Blood samples were obtained after an overnight fast. Valid data on vitamin D were available in 1,622 subjects (94%), 50.1% men and 49.9% women. Blood samples for vitamin D (25-OH-vitamin D) were stored at −80°C, and analysed concurrently when all participants were screened. The method used for S-25(OH)D was HPLC (LC–MS/MS), the golden standard method for vitamin D analyses (27–29). Analyses were performed at a laboratory ascertained through DEQAS external controls. The method was calibrated with Chromsystems (Munich, Germany) calibrator for 25-OH-vitamin-D3 and 25-OH-vitamin-D2. The calibrator was directly traceable to NIST (National Institute of Standards and Technology, Gaithersburg, MD, USA). The method was also controlled with NIST SRM 972 and was within assigned values for both 25(OH)D3 and 25(OH)D2 in all 4 levels.

The limit of detection was 6 nmol/L. The inter-assay coefficients of variation (CVs) were 3% at 25D3 level 88 nmol/l and 2.6% at 25(OH)D3 level 177 nmol/l. The intra-assay CVs were 2.3% at 25(OH)D3 level 18 nmol/l and 1.7% at 25(OH)D3 level 48 nmol/l. Both 25(OH)D3 (cholecalciferol) and 25(OH)D2 (ergocalciferol) were quantified separately, but 25(OH)D2 values were low and detected only in 16 participants and therefore not included in the analyses. For readability, results of 25(OH)D3 were named vitamin D3 or just D3 in the text. Levels used for definitions were the current levels in Sweden and in accordance with the Institute of Medicine; deficient, D3<25 nmol/l (<10 ng/ml); insufficient, D3 25–49.9 nmol/l (10–20 ng/ml); and adequate, D3≥50 nmol/l (20 ng/ml) (30,31). One participant was excluded as an outlier with 295 nmol/l.

### Statistical method

We report mean values and 95% confidence intervals of vitamin D3 levels for categorical variables and test for differences with ANOVA. Adjustment for age and gender was performed using univariate general linear models.

### Ethics

The 2009 MONICA population survey is covered by ethical permission from Umeå University, number 08–106M.

## Results

**Fig. 2:**
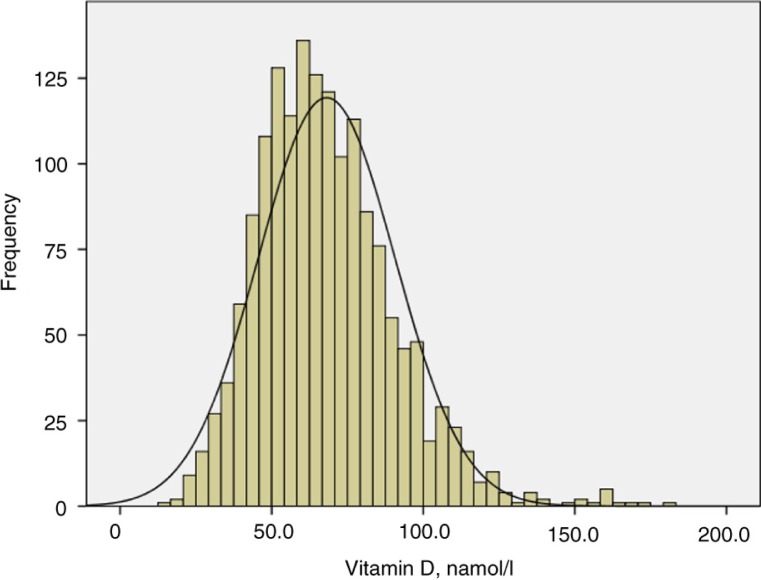
Distribution of vitamin D3 levels in the population of northern Sweden in 2009, aged 25–74 years.

The age and sex distribution among the participants is presented in Table I. The distribution of D3 in the population is presented in Fig. 2; values range from 15 to 180 nmol/l. The mean level was 68.1 nmol/l, corresponding to 65.2 and 71.0 nmol/l, in men and women, respectively (p<0.001). The median level was 66 nmol/l, corresponding to 63.6 and 67.7, in men and women, respectively. When excluding subjects using vitamin D supplements (n=218), the mean level decreased among men to 64.8 and among women to 69.3 nmol/l. There were no significant differences in mean or median D3 levels between the 4 months of sampling.

**Table I:** Age and sex distribution of participants in the Northern Sweden MONICA population survey 2009

		Sex	
		
		Men	Women
Age group	25–34	122	130
	35–44	159	169
	45–54	157	165
	55–64	184	173
	65–74	191	172
	Total	813	809

Adequate levels of D3 (≥50 nmol/l) were found in 79.2%, more so in women than in men (Table II, p<0.001). In men 23.1% were in the range defined as insufficient (D3 25.0–49.9 nmol/l) and in women 17.1%. Only 12 participants (0.7%) were D3-deficient (D3<25 nmol/l), 10 men and 2 women. There were no difference in the prevalence of D3 deficiency between months of survey and when excluding subjects using vitamin D supplements, prevalence of insufficiency did not change substantially.

**Table II:** Proportion of participants with deficient, insufficient or adequate levels of vitamin D3 according to gender (nmol/l)

		Deficiency (<25)	Insufficiency (25–49.9)	Adequate (≥50)
Sex	Men	1.2%	23.1%	75.6%
	Women	0.2%	17.1%	82.7%

**Fig. 3:**
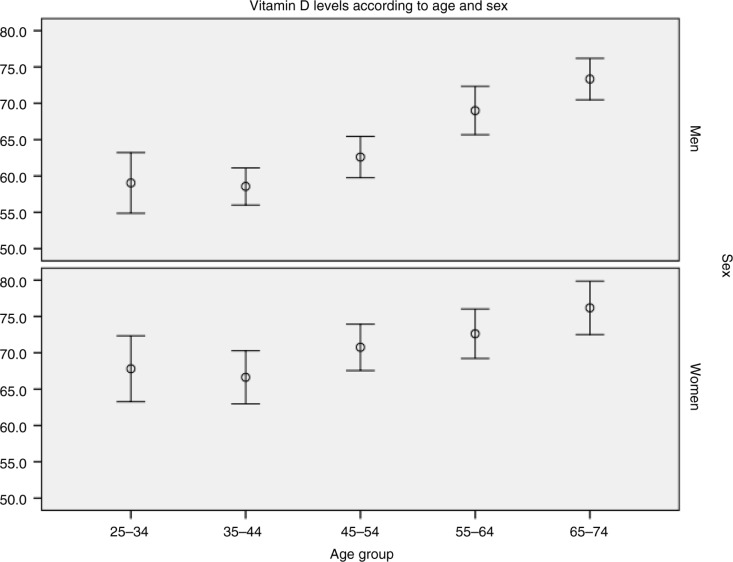
Mean levels of vitamin D3 in the population of northern Sweden in 2009 according to age, group and gender (bars mark 95% confidence intervals).

D3 levels increased with age (correlation r=0.19, p<0.001) and age group (Fig. 3, ANOVA linear test for trends, p<0.001 and p=0.002, for men and women, respectively). Insufficient levels were most common in the youngest age group (25–34 years), 41.0% in men and 22.3% in women.

There were no differences in D3 between the northernmost county of Norrbotten and the more southern county of Västerbotten, the 2 regions sampled in the Northern Sweden MONICA Study, nor were there any differences in urban versus rural living (data not shown). Subjects born outside of Sweden or Finland, especially from outside of Europe, had lower D3 than native Swedes and Finns (p<0.001), but foreign-born subjects were few (n=53, Finland excluded). D3 did not differ according to level of education.

The 218 participants who reported supplementation with vitamin D had a mean D3 level of 75.6 nmol/l compared to 67.0 in those who did not use supplementation. After age and sex adjustment, vitamin D supplementation remained strongly associated with higher levels of vitamin D (p<0.001).

## Discussion

Sun exposure is the most important source of vitamin D in some populations (32). People living in the sub-arctic area of Sweden receive few or no hours of sun during winter. The sun never rises more than 30° over the horizon during winter, which is the limit under which there is no vitamin D production at all. Despite these facts, we found that D3 deficiency is very uncommon even during winter and that young men are those who are most D3 insufficient, up to 40%. Insufficiency occurs in about 20% of the population aged between 25 and 74 years. Supplementation with vitamin D contributed little to population levels. Notwithstanding, D-vitamin supplementation was strongly associated with higher levels although rarely used in this population.

To facilitate a comparison with other studies, a literature review was performed searching for population-based studies on D3 levels in Sweden (9,11,13,14,20,33), Norway (6,10,12), Finland (8), Denmark (7), Iceland (15) and northern Russia (22) (Table III). Even though many studies are old and include narrow age ranges, our population mean of 68 nmol/l is clearly above the large Danish (7) and Norwegian studies (12). Although very high D3 levels were reported from a Swedish case-control study (13), these samples were from the 1980s and should be cautiously interpreted.

**Table III:** Studies from northern Europe on D3 levels (nmol/l) in the population sorted according to northern latitude

Study	Year	Area	Latitude	Season (month)	Number	Age	D3 mean total	D3 mean women	D3 mean men	Insufficient (%)
Kozlow (22)	2009–2012	N Russia	66–67N	Complete year	188	18–60	31–50			34–58
Brustad (6)	2002	N Norway	65–71N	Mo 11–5	443	44–59		57		38
This study	2009	N Sweden	63–69N	Mo 1–4	1,622	25–74	68	71	65	20.1
Sigurdsson (15)	1998	Iceland	64N	Mo 09–06	308	70		53		13
Larose (12)	1995–1997	Mid Norway	64N		2,505	19–55	59	59	58	40
Standahl Olsen (RW.ERROR – unable to find reference: 1,040)	2005	Norway	58–70N	Mo 4–8	372	48–62		42		77
Burgaz (9)	2006	Mid Sweden	60N	Mo 1–3	116	61–86		69		18
Deleskog (33)	1992–1998	Stockholm	60N	Complete year	2,022	35–56		57	61	
Lamberg-Allardt (8)	1998	S Finland	60N	Mo 2–3	328	31–43		47	45	
Littorin (13)	1987–1988	Sweden	55–69N		208	25	97	100	93	
Landin-Wilhelmsen (20)	1984	Gothenburg	57N	Mo 3–11	382	25–64		73	66	52.2
Macdonald (32)	2006	Scotland	57N	Complete year	377	55–70		28		76
Thuesen (7)	1999–2001	Denmark	56N	Complete year	6,146	30–60	48			
Shirazi (14)	1991–1996	Malmö	55N	Complete year	727	41–73		88		
Gerdhem (11)	1996–1999	Malmö	55N	Complete year	986	75		95		4

Vitamin D status has been extensively studied in Greenland over the past years and compared to people living in Denmark, both of Inuit and Danish origin (23,34–36), and were recently reviewed (37). Even though Greenlanders may have an enhanced renal production of vitamin D and an effective dermal production even at latitude of 70°N, a decrease in vitamin D levels has been found and explained by the less traditional Inuit diet.

Most studies focus on women, often post-menopausal, mirroring the previous narrow focus on bone health. Excluding the case-control study with young subjects (13), 2 studies from Malmö stand out with high levels among women (11,14) and very low prevalence of insufficiency. Otherwise most studies in women (6,8–10,12,15,20,33) are in the range of 42–73 nmol/l. Extending the geographical area to Scotland (latitude 57°N), D3 levels in women were as low 28 nmol/l (32). This leads us to conclude that among women in northern Sweden, the population mean of 71 nmol/l is high. This may possibly be explained by high consumption of fatty fishes, such as salmon, vitamin supplementation and sun bathing holidays during the winter although this was not measured in our study. We are well aware that direct comparison between studies is difficult due to separate assay methods, populations and secular trends, though.

The 4 cohort studies on men (8,12,20,33) all report similar levels, ranging from 45 to 66 nmol/l, well in accordance with men in MONICA 2009 having 65 nmol/l.

In Table III we sorted the studies according northern latitude but no clear trend in D3 according to a north–south gradient was found. However, among the most northerly studies is evident that northern Sweden has higher D3 levels than Tromsö (6), the HUNT study from Mid-Norway (12) and Iceland (15). That more northern latitude does not necessarily lead to lower D3 levels was evident in a European multicentre study, using similar sampling and assays, where D3 increased from Italy to Finland and Sweden (18). Perhaps this is due to dietary habits and vitamin D–supplemented food. In a large recent study from Greenland, higher latitude (ranging between 60 and 77°N) was associated with lower vitamin D levels (35) and dermal production of vitamin D during sun exposure contributes strongly (34).

In most studies, just as in MONICA, women have slightly higher levels of vitamin D. This may be due to more sunbathing (6), outdoor activities (6,7), sun holidays (9,17) and a more frequent use of vitamin D supplementation among women (9,14).

A recent review discussed the common finding of vitamin D deficiency among northern native people (21). However, it is important to keep in mind that although our study area is well within the circumpolar area, only approximately 5% of the inhabitants are of Sami descent (38). The lower levels we found among participants born in countries outside Scandinavia is also well known (17).

If there is no, or possible even an inverse, gradient between latitude and D3 in the population, both within Sweden and within Europe, how is this reconciled with the notion of sun exposure as the most important source of vitamin D? Northerners have lighter skin pigmentation, which allows greater vitamin D synthesis. Additional adaptions include sun-seeking behaviour and supplemented foods which could counterbalance the lack of sun. The finding of a north–south gradient in Greenland (35) raises the possibility that modern, mainly urban, living diminishes the influence of sun exposure.

In the MONICA Study, D3 levels increased linearly with age and insufficiency was common among men 25–34 years of age, which is at odds with the HUNT Study where no age dependency was reported (12). The MONICA Study is in accordance with a Finnish report on younger adults (8) and Swedish women (14). We did not evaluate the reason, but low dietary intake of D-vitamin, low use of D-vitamin supplementation and less travelling to sunnier regions among younger adults compared to older adults might contribute to this.

### Strengths and limitations

When interpreting these studies on vitamin D, many methodological considerations must be taken into account. Choice of assay used to analyse vitamin D gives largely diverging results (29). In contrast to many of the older studies, we used HPLC, the method seen as the gold standard (28), using a laboratory with external quality control. However, the definition of vitamin D deficiency is controversial (39,40) and Sweden lacks a consensus though the definition in this study is recommended (41). Many other studies have sampled throughout the year, but we collected our data between January and April so we probably measured the lowest values in the yearly variation (7). Using the truly randomly selected population sample from the MONICA Study gives further credibility that our results have a high validity.

Data on sun exposure, diet, and travelling habits would have improved our analysis attempting to explain these finding but our limited scope was primarily to study the prevalence of insufficiency or deficiency of vitamin D in a northern population during the darkest part of the year.

## Conclusion

The large majority of adults living close to the Arctic Circle in Sweden have adequate D3 levels even during the second half of the dark winter. Subjects with D3 deficiency were uncommon but insufficient levels were often found among young men. The causes of this must be the topic of further studies.
